# Wirelessly Powered Endoscopically Implantable Devices into the Submucosa as the Possible Treatment of Gastroesophageal Reflux Disease

**DOI:** 10.1155/2019/7459457

**Published:** 2019-04-07

**Authors:** J. Hajer, M. Novák, J. Rosina

**Affiliations:** ^1^2nd Department of Internal Medicine, Charles University, Third Faculty of Medicine, Prague, Czech Republic; ^2^Department of Medical Biophysics and Medical Informatics, Third Faculty of Medicine, Charles University, Prague, Czech Republic

## Abstract

Gastroesophageal reflux disease (GERD) is a rather common disease with a prevalence reaching up to 10 or 20% in the western world. The most specific symptoms which point to the diagnosis of GERD are feelings of heartburn and the regurgitation of acidic stomach contents into the esophagus. However, a certain number of patients do not respond to standard therapy, and in these cases, it is necessary to resort to other treatment methods, such as laparoscopic fundoplication or electrostimulation of the lower esophageal sphincter. The aim of our work was to design and manufacture a miniature, battery-less stimulator to provide electric stimulation of the lower esophageal sphincter, which could be implanted deep into the submucosa of the distal esophagus. The main goal was to provide a battery-less system as opposed to traditional battery neurostimulators to reduce the size and weight of the device. An electronic prototype of a wirelessly powered implantable device was developed. We used animal models for the experiments. The device is designed to treat GERD via electrical stimulation of the muscularis propria. It is implanted into the submucosal pocket by the lower esophageal sphincter with an endoscope. This method of implantation is superior to esophageal stimulators used today because of very low invasiveness of the surgery. Bipolar neurostimulation via two gold-plated leads is provided. The device does not have any source of energy; it is powered wirelessly which reduces the risk of potential battery leakage and reduces the overall dimensions.

## 1. Introduction

The gastrointestinal tract (GIT) nervous system is a complex, independent network of neurons and glial cells which is responsible for controlling the functions of the gastrointestinal tract, including its motility, secretory function, and its role in immunoregulation. This network is made up of small ganglia and neurons interconnected by bundles of nerve fibers, which run along the entire gastrointestinal tract. Interstitial Cajal cells, as well as neurons, are also an important part of the enteric neural system. These are nonglial cells which can be found inside the entire gastrointestinal tract. They function much like a cardiostimulator and produce electrical activity, which leads to a peristaltic motion of the intestine in the form of slow waves [1]. The lower esophageal sphincter is made up of smooth muscles and keeps its contraction due to neurological and myogenic factors. Recent studies [2–4] suggest that electric stimulation of the gastrointestinal nervous system may represent a significant benefit for patients suffering from disorders such as gastroparesis (being effective for more than 10 years [5]), GERD, and constipation, or those who are not responding to therapy [6]. GERD and/or dysphagia is prevented by a correctly functioning lower esophageal sphincter. The LES is controlled by paracrine, hormonal, and neural factors, and it as well as the diaphragmatic sphincter works to stop gastric contents being refluxed into the esophagus [7].

Although electrostimulation therapy of the lower esophageal sphincter is a relatively new concept for the treatment of patients who are resistant to medication and also, the therapy is safe and effective in short-term and long-term studies in humans [3, 4, 6, 8], there have been no negative side effects to this form of the treatment and it has been proven to provide both significant and sustained relief from the symptoms of GERD while at the same time eliminating the need for PPI medication and reducing esophageal acid exposure. Canine models were first used to study the effects of electrostimulation of the LES in the treatment of GERD [9, 10]. Reports have stated that electric stimulation (20 Hz, pulse width of 3 ms) with 2 pairs of electrodes causing a contraction and increase of the pressure of the sphincter complex was effective in preventing gastroesophageal reflux. The effects of electrostimulation of the LES in patients with GERD using both high (20 Hz, pulse width of 200 *μ*s) and low (6 cycles per minute, pulse width of 375 ms) frequencies have also been examined. Both high- and low-frequency electrostimulation increased LES pressure but did not affect LES relaxation or residual pressure when swallowing [2]. It has been shown that high-frequency stimulation is preferable as it requires less energy and therefore extends the life of the battery. There are only two GIT stimulators currently in use, the Enterra II [11] and EndoStim [12], which use intramuscular catheters to stimulate gastric muscle tissue. Both of these require surgical implantation under general anesthesia and have a large unwieldy unit attached. As such, the option of a device implanted into the gastric submucosal layer which communicates wirelessly would be a large step forward in patient comfort. Neurostimulation of LES using endoscopically implanted leads exteriorized transnasally was also assessed and was successful, resulting in significant increase in LES pressure with no complaints of dysphagia [4]. Research has already proven that it is possible to implant a miniature neurostimulator into the submucosa [13, 14]. This research provides a scope for further improvements regarding power management (especially the option of wireless power device without battery), conforming to the rules and regulations for medical implants and wireless communication and the possibility of bipolar neuroelectrostimulation.

## 2. Material and Methods

### 2.1. Implantable Device Prototype Construction

The device which was constructed to assess the technology consists of 4 main components—printed circuit board (PCB) with electrical components, wireless power receiving coil, liquid-resistant enclosure, and stimulation electrodes.

The main PCB is manufactured on a FR4 material and the thickness is 0.8 mm. The electronics comprises of two main parts—control and power management.

The control part is integrated into a single microcontroller—PIC16LF1783—which is used to generate the electrical stimulation impulses. Two timer modules are used to generate stimulation pulses—the first timer sets the frequency of pulses and the second timer is used to turn on and off the stimulation at predefined times. The pulses generated by the logic part of the microcontroller is then amplified by on-chip operational amplifier and outputted to the stimulation electrodes.

The power management circuitry contains 3 main parts—voltage doubler with Avago HSMS282P zero-bias Schottky diodes, parallel LC resonant circuit with receiving coil, and low-drop regulator. A 5.1 V Zener diode is placed across the rectified voltage to protect the capacitor bank against damage due to overvoltage. The rectified voltage is converted to a stable 2.5 V DC power rail with a TPS70625 low-drop voltage regulator. This power rail is used to power the microcontroller. TLV803 voltage supervisor is utilized to avoid undervoltage lockout condition.

The main PCB is protected from the surrounding space using a technique which is today used in implantable medical devices like breast implants—by coating with functional biopolymers. In this case, multiple dip-coating of skin-colored 3Dresyn-MF UV-cured monomer-free resin for 3D printing was used. Between each coating, a curing schedule of 1 minute of 500 mW/cm^2^ UV light with a wavelength of 405 nm from each side was performed. A total of 4 coatings were required to fully cover the device.

**Figure 1:**
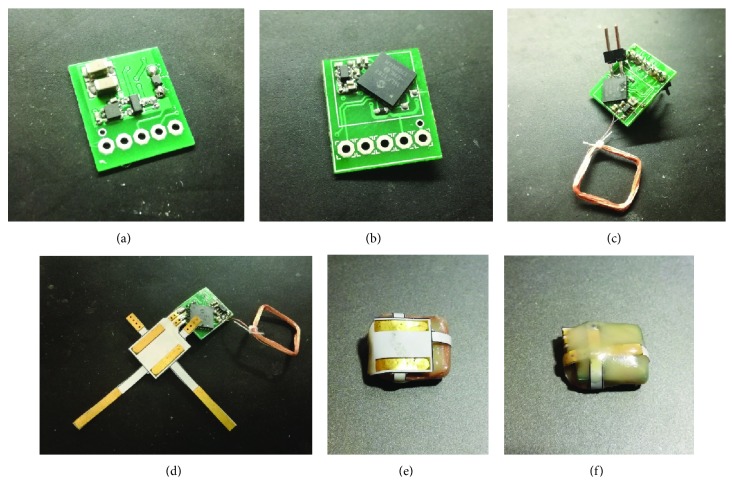
Composite picture of the implantable device prior to implantation: (a) back side of the PCB, (b) front side of the PCB, (c) PCB prepared for programming and testing, (d) trimmed PCB with stimulation electrodes ready for encapsulation, (e) encapsulated PCB—front side, and (f) encapsulated PCB—back side.

On the outside, the stimulation electrodes are connected. To reduce the thickness, the electrodes are manufactured on a polyimide substrate as a flexible printed circuit board. The electrodes are gold plated to limit corrosion and enhance biocompatibility. The electrodes are glued to the encapsulated electronics with the coil, and two straps are wound around the electronics and soldered on the other side, securing the electrodes against separation which occurred during first experiments. The completed device is depicted in Figure 1.

### 2.2. The Wireless Powering Device

The powering device was energised by an alternating magnetic field with a frequency of 1 MHz. This magnetic field was created by a custom-developed device intended for this task. This device comprised of a printed circuit board, a heatsink, and a rectangular coil composed of 3 turns. The coil was connected in series with a capacitor bank and tuned to a resonance frequency of 1 MHz. This was done to maximize the current flowing through the coil. The magnetic field strength in a constant distance from a wire is proportional to the current flowing through the wire.

(1) $$\begin{array}{c} B=\frac{\mu_{0}I}{2\pi r}. \end{array}$$

By measuring the impedance of the coil at target frequency, the resonance capacitor value was determined. The alternating current at predefined resonance frequency is then generated by an H bridge formed by four N-MOSFET transistors. The control signals for the MOSFET transistors are generated by a dedicated microcontroller.

### 2.3. Energy Propagation through Tissue

One of the major concerns in wireless power transfer is the influence of surrounding materials, especially materials in between a receiving and transmitting device. In this case, the energy is transferred via air coupling of a transmitting and receiving coil. This is commonly referred to as “near-field” communication. The second type of energy transfer is far-field which uses electromagnetic waves to transmit energy. The antenna size is then proportional to the wavelength. For 1 GHz, the wavelength in vacuum is around 30 cm. However, electromagnetic waves are significantly attenuated at these frequencies. The requirement of using high frequencies to achieve good antenna gain, attenuation by tissue, and regulatory requirements renders far-field energy transfer to wireless implant impractical.

**Figure 2:**
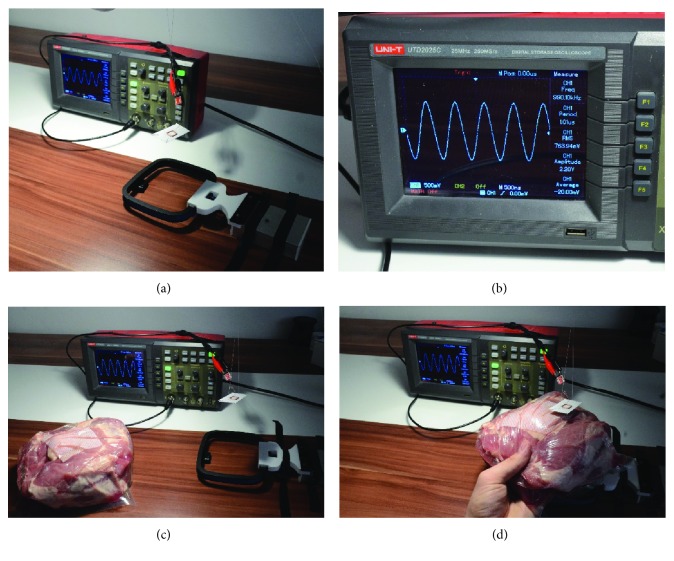
Composite picture of the experiment which evaluates effectivity of wireless power transfer through tissue: (a) measurement setup (receiving coil hovers 11 cm above transmitter coil), (b) detail of oscilloscope screen, (c) testing without the presence of porcine tissue, and (d) testing with the presence of porcine tissue.

The near-field wireless power transfer in this frequency range can be significantly affected only by materials with high conductivity by creating eddy currents in them (metals) or materials with high magnetic permeability (e.g., mu-metal or permalloy). To support this statement, an experiment was conducted (Figure 2). We have secured a wireless receiver coil with a parallel resonant capacitor and wireless transmitter 11 cm apart each other. The first measurement was done with no object placed between the coils. A 1 kOhm resistor was placed across the receiving coil resonant circuit to simulate an electric load. The voltage across the resistor with energy transfer active was measured, and received power was calculated using Ohm's law. After that, the experiment was repeated but in between transmitting and receiving coil, an 8 cm thick porcine tissue was placed. The average power (averaged over 10 seconds) received with and without animal tissue in between was 0.560 mW and 0.588 mW, respectively. This is in accordance with the theory that the effect of tissue on this type of wireless power transfer is minimal (4.7% decrease). One of the possible explanations of the decrease is detuning of the transmitting LC circuit. This may be compensated for during development, and the effect of the tissue presence will be further minimized (at the same distance and angular position of the coils, the power transferred will be smaller without the presence of the tissue).

### 2.4. Animal Model

**Figure 3:**
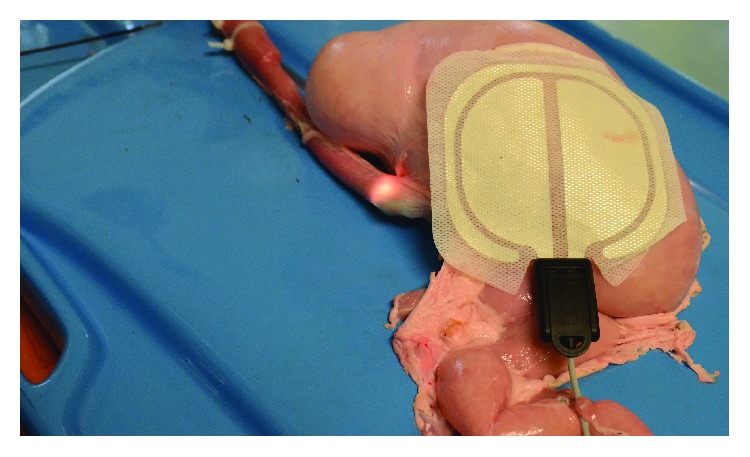
Animal model with the highlighted place of the implantation of the device near the lower esophageal sphincter.

A porcine model made of the stomach and a long segment of the esophagus was used. It is a commonly used model for training of techniques such as ESD (endoscopic submucosal dissection), tunnelisation, and POEM (peroral endoscopic myotomy). The overall view of the model with the implanted device and inserted endoscope is provided in Figure 3.

### 2.5. Endoscopic Implantation of the Device

**Figure 4:**
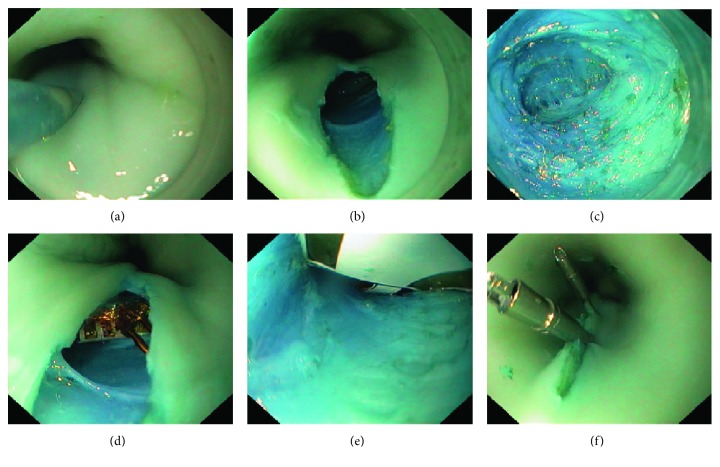
The process of implanting the device as shown in a composite picture: (a) submucosal injection; (b) vertical opening; (c) view of submucosal tunnel; (d) device inside the tunnel; (e) final implant positioning; (f) opening closure.

Using the same endoscopic submucosal tunnelling method usually used for POEM, first described by Inoue et al. [15], the device was implanted into the submucosa. This procedure is documented in Figure 4. A combination of methylene blue and saline solution is first injected about 5 cm above the LES into the submucosal layer with a therapy needle catheter (25G). An electrosurgical knife is used to make an opening into the submucosa. This submucosal pocket is then dilated and disrupted, thus creating a 5 cm long tunnel large enough for the implantation of the device. Using a grasper, the device is moved into the area of the pocket and released. Grasping forceps then move the device into the submucosal tunnel. The opening made by the initial incision is then closed with haemostatic clips.

**Figure 5:**
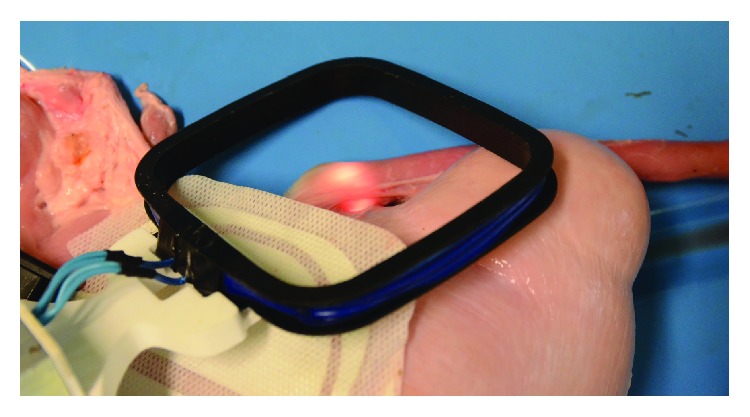
Powering the implant using wireless inductive power transfer.

After implantation, a transmitter coil, which produces an alternating magnetic field of 1 MHz frequency, is powering the implantable device (Figure 5).

## 3. Results

The prototype of the esophageal neurostimulator was successfully endoscopically implanted in a pig model. We used the tunnelisation method. The prototype was attached in the vicinity of the muscular layer of the LES. The entire procedure took approximately 30 minutes in total and was without any perforation or other complications. The device and its functions were tested with an oscilloscope ex vivo.

**Figure 6:**
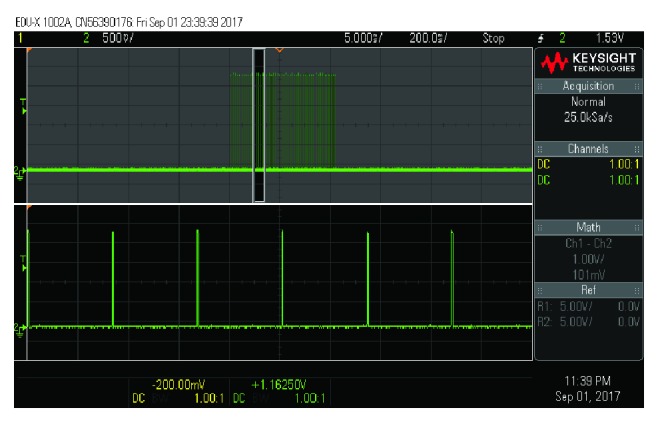
Stimulation pattern waveform generated by the implantable neurostimulator.

The wireless energy transfer device was successfully able to power the implant from approx. 12 cm. This means that the microcontroller in the device was able to power up correctly and start generating stimulation patterns (Figure 6).

Next, the presented design of electrodes does not separate from the device which was one of the main issues during previous experiments. The electrodes are also constructed from intrinsically biocompatible materials (polyimide and gold, respectively).

A novel method of dip-coating of the device in biocompatible monomer-free resin was used which is a major improvement over previous research which did not use biocompatible coatings for device prototypes.

The weight of the neurostimulator is 1.22 grams (60% decrease over the previous experiment), and the volume is 0.74 cm^3^ (40% decrease over the previous experiment).

## 4. Discussion

This test proves that a tiny implantable device without a battery may be used for LES neurostimulation. This innovative neurostimulator could provide patients with a reliable and comfortable solution to currently used surgical methods. The device has very low power requirements in standby, in terms of tens of microwatts, because it has no wireless communication. Through power cycling of the energising coil externally, the rate of neurostimulation can be controlled.

Endoscopically implanted battery-less devices which control neurostimulation have potential uses not only in the general population but also in problems caused by other sphincter dysfunctions. Although endoscopically implanted electrodes are proven to be effective [8], the determination of the efficacy of the neurostimulator on live animals will require further experimentation to be confirmed. Based on previous experiments with implantation of a device to the stomach and esophagus, we have found a size limit of the device. This was the primary motivation for the development of battery-less version of the device. The battery and charging electronics form a significant portion of the volume of the device. Also, any battery always represents a hazard, when any explosion or leakage in this specific area could result in serious injury or death. Thus, putting the energy source outside of the implant was a logical step to reduce the size and increase safety. In this experiment, we have confirmed that this topology of an implantable neurostimulator is feasible.

The new method of creating a biocompatible housing around the device is suitable for short-term experiments. When performing longer experiments (i.e., weeks), there is a possibility that moisture could leak into the implant via the interface between the PCB and outside of the implant where the stimulation electrodes are located. In that case, a layer of conformal coating of the PCB before coating the PCB with biocompatible 3D printing resin could add sufficient protection. In the case of a not biocompatible material, there is a significant risk of implant rejection. Also, the implant could be prone to migration, requiring additional solution for fixation.

## 5. Conclusions

This research has proven that the lower esophageal sphincter can receive controlled neurostimulation from a miniature implantable device without a battery. The neurostimulation can be provided by our solution which makes a relatively simple and, most importantly, reliable device. Its wireless nature means that it has very low power needs, only tens of microwatts. By power cycling the energy coil externally, we can regulate the power and rate of neurostimulation.

This technology presents a promising option for use in the general public with such problems as GERD. In both cases, the size of the device, its ease of implantation, its longevity, and its safety offer a leap forward when compared with contemporary neurostimulation solutions. On the other hand, the endoscopic implantation is quite a challenging procedure comparable to POEM. Our opinion is that the implantation procedure is easier because it does not require myotomy. But in almost every country, a high-volume centre for POEM is present. Thus, the accessibility of the treatment should be high. Periprocedural complications like bleeding and perforation can occur. On the other hand, data which supports high safety of POEM procedure is available [16]. On the other hand, fundoplication which was examined as a possible solution for GERD has worse track record according to literature [17].

Based on these results, we plan to confirm the effect of the stimulation of the device on a living pig with an esophageal manometry. For these experiments, it is planned to make a special enclosure for biocompatible materials as the device is expected to stay in the submucosa for extended durations of time (at least several weeks). The enclosure will be either machined from biocompatible polymer (i.e., PEEK) or made using additive manufacturing from medical-grade resins. The position of the neurostimulator close to the lower esophageal sphincter creates an opportunity to place a pH sensor outside of the submucosa. A feedback-controlled neurostimulator which would use real-time data from a pH sensor to control the neurostimulation could offer significant power savings as the stimulation would be active only when a reflux episode occurs.

## Data Availability

The detailed description of the hardware as well as the implantation technique used is described in the article. The images which demonstrate successful implantation of the device into the submucosa and ex-vivo test of the implantable device (which are the results of the research) are also included within the article.
